# Characterisation of the potential function of SVA retrotransposons to modulate gene expression patterns

**DOI:** 10.1186/1471-2148-13-101

**Published:** 2013-05-21

**Authors:** Abigail L Savage, Vivien J Bubb, Gerome Breen, John P Quinn

**Affiliations:** 1 Department of Molecular and Clinical Pharmacology, Institute of Translational Medicine, The University of Liverpool, Liverpool L69 3BX, UK; 2 King’s College London, MRC Social Genetic and Developmental Psychiatry Research Centre, Institute of Psychiatry, London, UK; 3 National Institute for Health Research (NIHR) Biomedical Research Centre for Mental Health, South London and Maudsley NHS Foundation Trust and Institute of Psychiatry, King’s College London SE5 8DF, UK

**Keywords:** SVA, PARK7, DJ-1, Genetic variation, G-quadruplex DNA, Retrotransposon

## Abstract

**Background:**

Retrotransposons are a major component of the human genome constituting as much as 45%. The hominid specific SINE-VNTR-Alus are the youngest of these elements constituting 0.13% of the genome; they are therefore a practical and amenable group for analysis of both their global integration, polymorphic variation and their potential contribution to modulation of genome regulation.

**Results:**

Consistent with insertion into active chromatin we have determined that SVAs are more prevalent in genic regions compared to gene deserts. The consequence of which, is that their integration has greater potential to have affects on gene regulation. The sequences of SVAs show potential for the formation of secondary structure including G-quadruplex DNA. We have shown that the human specific SVA subtypes (E-F1) show the greatest potential for forming G-quadruplexes within the central tandem repeat component in addition to the 5’ ‘CCCTCT’ hexamer. We undertook a detailed analysis of the *PARK7* SVA D, located in the promoter of the *PARK7* gene (also termed *DJ-1*), in a HapMap cohort where we identified 2 variable number tandem repeat domains and 1 tandem repeat within this SVA with the 5’ CCCTCT element being one of the variable regions. Functionally we were able to demonstrate that this SVA contains multiple regulatory elements that support reporter gene expression *in vitro* and further show these elements exhibit orientation dependency.

**Conclusions:**

Our data supports the hypothesis that SVAs integrate preferentially in to open chromatin where they could modify the existing transcriptional regulatory domains or alter expression patterns by a variety of mechanisms.

## Background

Mobile DNA, such as long terminal repeats (LTRs), long interspersed elements (LINEs), short interspersed elements (SINEs) and SINE-VNTR-Alus (SVAs), constitute up to 45% of the human genome. These retrotransposable elements are mobilised via a ‘copy and paste’ mechanism; namely a RNA intermediate is reverse transcribed into DNA which inserts back into the genome at a different loci to the source sequence. Historically SVAs were originally identified as a sequence derived from part of the *env* gene and a 3′LTR from the HERV-K10 endogenous retrovirus with a poly A-tail and a GC-rich tandem repeat directly upstream and were named SINE-R elements [1]. It was later shown that in the C2 gene, the GC tandem repeat of the SINE-R element was a variable number tandem repeat (VNTR) [2]. This composite element was termed a SINE-VNTR-Alu (SVA) when further analysis of its components revealed the Alu-like sequences adjacent to the VNTR [3]. Thus typically SVAs consist of a hexamer repeat (CCCTCT), an Alu-like sequence, a GC-rich VNTR, a SINE and a poly A-tail.

Such SVAs, which are hominid specific, are to date the smallest of the retrotransposon families identified with 2676 elements found in the Hg19 amounting to 0.13% of the genome. A precursor of the VNTR domain found within the SVAs is present within the rhesus macaque genome, many of these precursor elements are also present in the human genome suggesting they were retrotransposing prior to the divergence of the old world monkeys and the hominoids [4]. SVAs are divided into subtypes (A-F) by the SINE region and their age estimated at 13.56Myrs old for the oldest subtype (A) and 3.18Myrs old for the youngest subtype (F) [5]. A seventh subtype has been identified that contains a 5’ transduction of the sequence from the first exon of the MAST2 gene and associated CpG island and has been referred to as either CpG-SVA, MAST2 SVA or SVA F1 [6-8]. The sequence of the MAST2 loci that has been incorporated into the F1 structure has been shown to act as a positive regulator of transcription in a reporter gene construct when transfected into human germ cells and is thought to have contributed to the success of the subtype in its retrotransposition [9]. Subtypes E, F and F1 are human specific as are some members of SVA subtype D. The younger subtypes appear to contain two GC rich VNTRs as opposed to the one seen in the older subtypes.

SVAs are non autonomous and are mobilised by the LINE-1 protein machinery [10,11], their retrotransposition rate is estimated at 1 in every 916 births [12]. A recent study to determine the nature of SVA retrotransposition revealed that no individual domain of an SVA is fundamental for this to occur, but each domain differentially affected the rate at which retrotransposition can take place [13]. To date eight SVA insertions have been associated with disease [14,15], these include for example a SVA in the 3’UTR of the *fukutin* gene which causes Fukyama-type congenital muscular dystrophy by decreasing mRNA production, and a SVA insertion and subsequent 14 kb deletion of the *HLA-A* gene locus linked with leukaemia [16,17]. Retrotransposition events are repressed in somatic cells via epigenetic modifications and post transcriptional suppression but there is recent evidence for these events occurring in the adult brain and their insertions are associated with protein coding genes active in the brain [18]. In tumour cells, SVAs along with other retrotransposons become demethylated and potentially could lose the epigenetic modifications that silenced them [19]. The latter indicates that retrotransposons including SVAs could modify the genomic structure of a locus with associated consequences for regulation without the requirement for retrotransposition.

The nature of the sequence contained within SVAs shows the potential for formation of secondary structures such as cruciforms and G-quadruplexes (G4) [20]. G4 DNA is a secondary structure predicted from bioinformatic analysis to form in guanine-rich sequences, but validation *in vivo* is difficult and highly debated [21-23]. G4 structures are hypothesised to interfere with replication of DNA and be involved in a host of regulatory functions including gene expression, genome stability and telomerase activity [24-27].

SVAs contain large domains of repetitive DNA (VNTRs) similar in copy number and size of individual repeats to those, we and others, have found to direct differential tissue specific and stimulus inducible gene expression in many other genes [28-35]. This differential regulator property has been correlated with copy number of the VNTR in some genes [30,34-43]. For example, we and others have demonstrated that VNTRs located in the promoter and second intron of the human serotonin transporter gene (*SLC6A4*) are differential as both risk factors for mental health and tissue specific regulators in the context of reporter gene constructs, *in vivo* and *in vitro* based on the copy number of the repeat [29,31,35].

In this communication we have determined the global location of SVAs, and then focused on the individual variation and function of a single selected SVA located in the *PARK7* gene promoter. The *PARK7* SVA was chosen because it is human specific, was regarded as a complete SVA and because of the nature of its location in relation to the *PARK7* gene. We addressed the potential function of this SVA as a transcriptional regulator, by investigating its activity in a reporter gene construct.

## Results

### Distribution of SVA elements across the human genome

**Figure 1:**
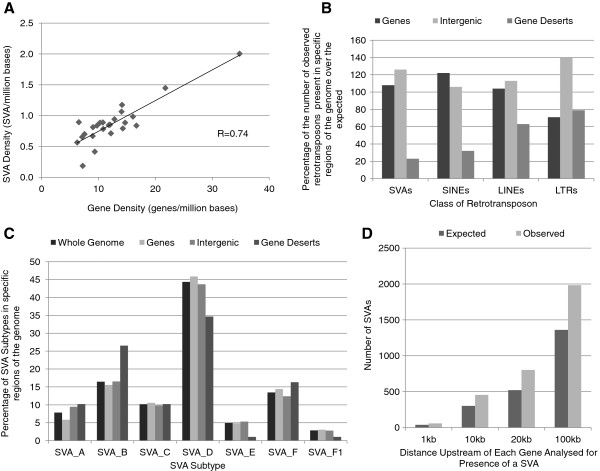
**Distribution of SVAs is associated with genic regions. A** - The SVA density of each human chromosome was plotted against the gene density of that chromosome showing a positive relationship between the two variables (correlation coefficient = 0.74). The correlation coefficient was calculated using bootstrap confidence interval (95%). **B** – The number of observed retrotransposons in defined regions of the human genome compared to the expected (based on the size of the region) and expressed as a percentage. (SVAs *X*^2^ = 339.5, df = 2, P < 0.001, SINEs *X*^2^ = 170647, df = 2, P < 0.001, LINEs *X*^2^ = 44320, df = 2, P < 0.001, LTRs *X*^2^ = 77018, df = 2, P < 0.001). **C** – The distribution of SVAs within genes, intergenic regions and gene deserts broken down by subtype and compared to their distribution across the whole human genome. (Genes *X*^2^ = 0.71, df = 6, P = 0.99), (Intergenic *X*^2^ = 0.47, df = 6, P = 0.99), (Gene deserts *X*^2^ = 13.91, df = 6, P < 0.05). **D** – The number of SVAs located within set distances upstream of a transcriptional start site (1 kb, 10 kb 20 kb and 100 kb) (*X*^2^ = 506.8, df = 3, P < 0.001).

The SVA density of each chromosome was found to be positively correlated with gene density (r = 0.74) as shown in Figure 1A (for values for each chromosome see Additional file 1). The correlation coefficient for the relationship between gene density and SVA density was calculated using the bootstrap confidence interval (95%) to remove outliers. However when the density of each SVA subtype was analysed individually, a negative correlation with gene density across chromosomes was found for subtype A, whereas all other subtypes showed a positive correlation (see Additional file 2). The oldest of the subtypes, A, show a clear difference in the pattern of insertion in the genome to the rest of this family of retrotransposons, however the mechanism behind this is unclear.

To dissect the distribution of SVAs further, the genome was divided into the three following regions: genes, intergenic and gene deserts and the observed distribution of members of the four classes of retrotransposon (LTRs, LINEs, SINEs and SVAs) was compared to the expected. The expected number of each element was determined by calculating the number of elements that would be present in the region in relation to its size if the elements inserted randomly throughout the genome. Gene deserts were defined as regions between genes which were 250 kb away from the start or end of a known gene, intergenic as regions between genes that are less than 250 kb from the start or end of a known gene and genes were determined by the UCSC gene track from the UCSC genome browser. The regions were defined in this manner to assess if the retrotransposons had preferentially inserted into regions devoid of genes (gene deserts) or regions of the genome that could include active chromatin where genes and intergenic regions potentially containing regulatory domains (up to 250 kb from TSS) are located [44,45]. The distribution of the different classes of retrotransposons shared some similarities, in particular a lower number than expected were found in gene deserts and all classes showed a significant difference in their actual distribution to the expected across the three regions analysed, Figure 1B (SVAs *X*^2^ = 339.5, df = 2, P < 0.001, SINEs *X*^2^ = 170647, df = 2, P < 0.001, LINEs *X*^2^ = 44320, df = 2, P < 0.001, LTRs *X*^2^ = 77018, df = 2, P < 0.001). The distribution of SVAs was further analysed by subtype within the previously defined regions: genes, intergenic and gene deserts (Figure 1C). The SVA subtypes showed a significant difference in their distribution within gene deserts compared to the whole genome (Gene deserts *X*^2^ = 13.91, df = 6, P < 0.05) but not within genes and intergenic regions (Genes *X*^2^ = 0.71, df = 6, P = 0.99, Intergenic *X*^2^ = 0.47, df = 6, P = 0.99). Subtypes D, E and F1 were underrepresented in gene deserts whereas subtype B in particular was found in higher numbers. The SVAs also showed a significant increase in regions 1-100 kb directly upstream of transcriptional start sites when the observed number was compared to the expected for the size of these regions (*X*^2^ = 506.8, df = 3, P < 0.001) (Figure 1D). The subtype distribution was significantly different within the first kilobase upstream of the start of transcription (see Additional file 3); subtypes A, B and E were found in lower numbers than expected and there were a greater number of subtypes C and D.

### Potential of SVA subtypes to form G-quadruplexes

**Figure 2:**
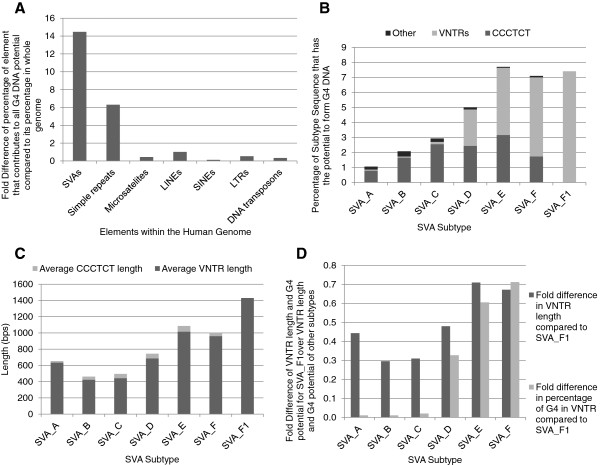
**The primary sequence of SVAs has the potential to form G-quadruplex DNA. A** – Potential G4 DNA formation was analysed *in silico.* The fold difference in the relative contribution of each element to their proportion in the whole human genome was calculated and is displayed. **B** - The percentage of sequence from each SVA subtype that could potentially form G4 DNA in the human genome according to Quadparser software is shown; it was further sub-divided into the following elements: CCCTCT hexamer repeat, VNTRs and the remainder of the sequence (other). **C** – Illustrates the relationship between VNTR and hexamer repeat length during evolution of the SVA subtypes. The average lengths are shown in base pairs. **D** – The fold difference in size of each of the central VNTRs from the SVA subtypes in the human genome, and their percentage contribution to form G4 compared to the value for SVA subtype F1 which has the highest value for both central VNTR length and G4 potential of the central VNTR.

**Figure 3:**
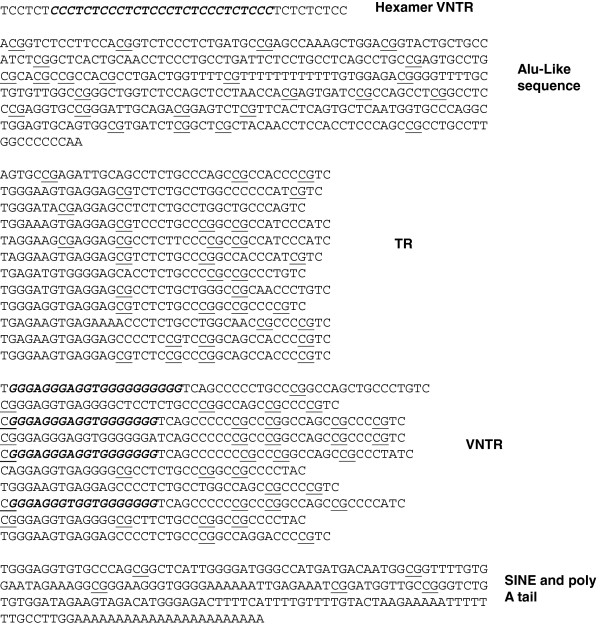
**Primary sequence of allele *1 *of *PARK7 *SVA identifying the different components.** The human-specific *PARK7* SVA located 8 kb upstream of the *PARK7* gene (chr1:8012112–8013618) contains a CCCTCT hexamer VNTR, Alu-like sequence, TR, VNTR, SINE and poly A-tail. In italics are the sequences of DNA that have been predicted to have the potential to form G4 DNA by Quadparser software, potential sites of methylation (CpGs) are underlined.

We investigated the potential of SVAs, more specifically the CCCTCT hexamer repeat at the 5’ end and the more central VNTR region, to form G4 DNA. Of the total genomic DNA that can form G4 DNA (predicted by Quadparser software [46]) 1.88% is due to SVAs which only constitute 0.13% of the human genome. When repetitive or mobile DNA elements, which include simple repeats, microsatellites, LTRs, LINEs, SINEs and DNA transposons (as defined by UCSC genome browser Hg19 http://genome.ucsc.edu/index.html) are compared; SVAs have the greatest potential contribution to G4 DNA for their size for any specific element (Figure 2A). The sequence of the *PARK7* SVA is shown in Figure 3 with the bases that contribute to its G4 potential predicted by Quadparser software in italics.

It was found that the percentage of sequence in each SVA subtype with the potential to form G4 increased as the age of the subtype decreased, thus subtypes E, F and F1 have the greatest potential for G4 formation (Figure 2B). This can be explained by the increase in the potential of the central VNTR region to form G4 DNA from subtype D through to F1. The possible amount of G4 formed by the CCCTCT repeat was found to increase through subtypes A to E; however the proportion it contributed to the total G4 potential of each subtype decreased. Subtype F1 does not contain a CCCTCT repeat therefore all of its G4 potential is within the central VNTR.

The average number of repeats in the CCCTCT domain varied between subtypes (Figure 2C) which accounts for the difference in G4 potential between the SVA subtypes in this particular domain; the longer the CCCTCT domain the greater the G4 potential. The average length of the GC rich VNTRs also varied between subtypes but length did not show the same direct correlation with G4 potential as in the CCCTCT domain. For example the VNTRs of subtype A are just under half the length of those of subtype F1, however they have only a hundredth of the potential to form G4 DNA when compared to the VNTR sequences of subtype F1 (Figure 2D). It appears that the subtypes fall into two main groups when analysing the G4 potential in the VNTRs. Subtypes A, B and C have very low G4 potential in their VNTRs compared to subtypes E, F and F1 with subtype D bridging the difference between the older hominid specific and younger human specific subtypes. This can be explained by the development of the additional second VNTR of the younger subtypes with differences in the primary nucleotide content to the first VNTR containing sequences that have the potential for G4 DNA (Figure 3).

### Genetic variation of *PARK7* SVA

**Figure 4:**
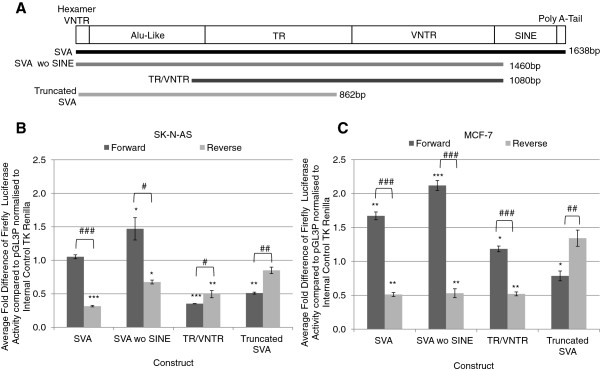
**The *PARK7 *SVA showed the ability to affect expression in a reporter gene construct. A** – Schematic showing the genomic structure of the *PARK7* SVA and the relationship to the fragments tested in the reporter gene constructs. **B** - The average fold activity of the different fragments from the SVA tested in both forward and reverse orientation over the minimal SV40 promoter alone (pGL3P) in the SK-N-AS cell line. Data was normalised to compensate for transfection efficiency, N = 4. **C** - The average fold activity in the MCF-7 cell line of the different fragments of the SVA in forward and reverse orientation over the minimal SV40 promoter alone (pGL3P) normalised to the internal control to account for transfection efficiency. N = 4. One tailed *t*-test was used to measure significance of fold activity of *PARK7* SVA fragments over SV40 minimal promoter alone (pGL3P) and to compare fold activity of forward and reverse orientations. * P < 0.05, **P < 0.01, ***P < 0.001, # P < 0.05, ## P < 0.01, ### P < 0.001. N = 4.

We analysed in detail the primary sequence and repeat variation in the human specific SVA D found upstream of the *PARK7* gene. The *PARK7* SVA is located 8 kb upstream of the *PARK7* major transcriptional start site defined by both the UCSC browser (http://genome.ucsc.edu/index.html Hg19) and the literature [47]. A putative alternative *PARK7* transcript also exists, that would originate within 1 kb of this SVA based on expressed sequence tags and other data in the UCSC browser and Archive ensembl (ensembl10:Jan2013). Genotypic analysis of this SVA identified four distinct alleles which were polymorphic in length, in 87 individuals from the CEU (Utah residents with Northern and Western European ancestry from the CEPH collection) HapMap cohort with allelic frequencies shown in Table 1. Alleles *1* and *3* were the most common within this cohort with 94% of the individuals having at least one of these alleles. The primary sequence of allele *1* of the *PARK7* SVA is shown in Figure 3 with the different domains, VNTRs, SINE and Alu-like, identified. Figure 3 also shows the CpGs underlined and the bases that contribute to the *PARK7* SVA’s G4 potential in italics. Allelic variation was found to be generated by differences in the number of repeat units present of specific repetitive elements within the SVA, namely the CCCTCT hexamer repeat and in the most 3’ of the two large central VNTRs. VNTR variation within the cohort was analysed by PCR and confirmed by a more limited sequence analysis of specific variants. The hexamer domain was either a 7, 10 or 13 repeat domain, and the second VNTR consisted of either 10, 11 or 12 repeats with a repeat length of 37-52 bp in this cohort. We observed no variation in the number of repeats in the most 5’ of the central ‘VNTRs’, which was a stable 12 copy variant of 37-40 bp repeat length, which was therefore termed a tandem repeat (TR). Schematic in Figure 4A shows the structure of the complete *PARK7* SVA and the variation found in its repetitive regions is summarised in Table 2.

**Table 1:** **Frequency of each allelotype for the *PARK7 *SVA in the HapMap cohort**

**Genotype**	**Number of samples**	**Percentage of allelotype within Hap Map samples**
*1/1*	19	21.8
*1/2*	4	4.6
*1/3*	35	40.2
*1/4*	4	4.6
*2/2*	4	4.6
*2/3*	3	3.4
*2/4*	1	1.1
*3/3*	16	18.4
*3/4*	1	1.1
*4/4*	0	0.0
Total	87	

The alleles are numbered 1–4 from shortest to longest.

**Table 2:** **Sequence analysis of the four alleles identified in the *PARK7 *SVA**

	**Number of repeats**		
**Alleles of *PARK7 *SVA**	**Hexamer VNTR**	**TR**	**VNTR**
1	7	12	10
2	10	12	11
3	10	12	12
4	13	12	12

Genomic DNA from individuals in the CEU (Utah residents with Northern and Western European ancestry from the CEPH collection) HapMap cohort was analysed. The length variation detected occurred in the CCCTCT hexamer repeat (termed hexamer VNTR) and within a second repetitive VNTR region further downstream. In this cohort a repetitive domain here termed a TR was not found to vary between the individuals within this population; this TR was located upstream of the second VNTR. The alleles were numbered 1–4 from shortest to longest. One example of each type of allele was sequenced.

### Functional activity of *PARK7* SVA in reporter gene analysis

We addressed whether both the intact *PARK7* SVA and its distinct individual domains could act as transcriptional regulators. SVAs can be found in the same, or opposite orientation to the gene they are located near to. When analysed, 49% of the SVAs found within 10 kb upstream of transcriptional start sites were on the same strand as the gene, for these reasons we also tested whether their function was orientation dependant. Eight reporter gene constructs were generated (Figure 4A) containing the following fragments in both forward and reverse orientations:

• the whole SVA (SVA)

• SVA with the SINE region deleted (SVA wo SINE)

• central TR and VNTR (TR/VNTR)

• a 5’ truncation with only the CCCTCT hexamer, Alu-like sequence and 10 of the 12 repeats of the TR of allele *1* of the *PARK7* SVA (truncated SVA) present

SVAs are described as having a CCCTCT domain at their 5’ end and a poly A-tail at their 3’ end therefore this was used to define the forward orientation. We compared the ability of the eight fragments to support reporter gene expression (luciferase) directed by a heterologous minimal promoter in two cell lines SK-N-AS, a human neuroblastoma cell line and MCF-7, a human breast cancer cell line.

In the SK-N-AS cell line (Figure 4B) the intact *PARK7* SVA in forward orientation did not alter the levels of reporter gene expression, when compared to the minimal promoter alone (pGL3P) however when the SINE domain was deleted reporter gene activity was significantly enhanced (p < 0.05). The TR/VNTR and the truncated SVA in the forward orientation acted to significantly repress luciferase activity when compared to the minimal promoter alone (pGL3P) (p < 0.001, p < 0.01 respectively). When the domains were tested in the reverse orientation the reporter gene levels were all significantly different when compared to the levels seen in the forward orientation (SVA p < 0.001, SVA wo SINE p < 0.05, TR/VNTR p < 0.05, truncated SVA p < 0.01). The activity of the SVA and SVA wo SINE in reverse orientation were reduced compared to when in the forward orientation whereas the activity of the TR/VNTR and truncated SVA showed the opposite trend.

The reporter gene constructs showed distinct activity levels in the MCF-7 cell line when compared to that observed in the SK-N-AS cell line (Figure 4C). In forward orientation the complete SVA had a significant increase in reporter activity in MCF-7 cells (p < 0.01), distinct from its function in SK-N-AS, however similarly to SK-N-AS cells the SVA wo SINE showed the greatest ability to enhance reporter gene activity. In contrast the TR/VNTR showed similar activity to that of the minimal promoter alone. The truncated SVA acted as a repressor as it did in the SK-N-AS cell line (p < 0.05). The domains in the reverse orientation all showed a significant difference to the activity of the domains in the forward orientation (SVA p < 0.001, SVA wo SINE p < 0.001, TR/VNTR p < 0.001, truncated SVA p < 0.01). The SVA, SVA wo SINE and TR/VNTR all showed decreased activity in the reverse orientation when compared to the domains in the forward orientation. The truncated SVA showed greater activity in the reverse orientation than when in the forward orientation.

## Discussion

Retrotransposons, including SVAs, can affect gene function by multiple mechanisms particularly when inserted into protein coding regions [48,49]. They have also been suggested to modulate transcriptional and post-transcriptional parameters based partially on their location within introns and promoters, however the functional significance of these non coding integrations is much more difficult to determine than those in exons. Epigenetic silencing which suppresses retrotransposition in somatic cells might have modulator effects on transcriptional or post transcriptional domains adjacent to sites of integration. Removal of such epigenetic silencing may correlate with retrotransposition in the aging CNS [18] and the observed hypomethylated state of SVAs in cancer [19]. This may suggest the potential for a dynamic chromatin structure over the locus of the SVAs under specific environmental conditions and challenges. In either circumstance the SVAs have the potential to influence the local genome architecture via epigenetic modifications, the formation of secondary structures and the binding of sequence specific transcription factors to the SVA.

Using the most recent version of the human genome, Hg19, we have demonstrated a minimum of 2676 SVA insertions in the human genome. This is considerably less in number than seen in the other classes of retrotransposons; this can be explained by the fact that they are the most recent family to integrate and proliferate in the genome. It is also likely that the primary DNA sequence of the members of this family has undergone the least number of alterations which may also suggest SVAs share related biochemical and functional properties. These properties will in part be directed by the primary sequence of the SVA to allow for such as interaction with transcription factors and other modulators of genome function acting as sequence specific binding proteins. A further regulatory function of the SVA could be directed by the genomic structure adopted upon insertion. Superimposed on these regulatory parameters could be modulation of their activity by the polymorphic nature of the distinct domains within the SVA such as the VNTR elements. There is an extensive literature on VNTR domains both being differentially associated with disease and transcriptional properties based on the copy number of the repeats [29,50]. In this study we addressed firstly the site of integration of SVA elements, secondly the potential secondary structures formed and finally a detailed analysis of the *PARK7* SVA’s ability to support reporter gene expression and its polymorphic nature. These are properties that would not only be involved in changing the transcriptome of a cell in disease states such as cancer, but also potentially a major driving force in evolution of the hominids.

We have characterised a preferential insertion of SVAs into genic regions (Figure 1), which may reflect the more accessible and open nature of the chromatin to allow for transcription and therefore more amenable to retrotransposon insertions than inactive chromatin. This is reflected in the finding that 62% of SVAs are within genes or their 10 kb flank. Waves of SVA retrotransposon integration in the hominids could alter significant number of genes via transcriptional/post transcriptional mechanisms which could act to initiate distinct cascades of gene expression changes which may have major phenotypic affects on cell function. There were also a greater number of SVAs than expected in key regions of the genome such as promoters (Figure 1D), these insertions have placed them where they could potentially influence transcription. The analysis of the prevalence of SVAs upstream of TSS was used to determine that throughout potentially regulatory regions of the genome SVAs are overrepresented. The CG-rich nature of the primary sequence of the SVAs [5] provides potential regions for methylation, many SVAs are located near the transcriptional start site of genes, therefore the methylation status of these elements could influence the expression of the gene as hypothesised for cancer [19,51,52]. Throughout the SVAs, their subtypes and domains share similar primary sequences; which provides the potential for binding similar sequence specific binding factors that could affect aspects of transcription or post transcriptional processing. The end result could be subsets of SVAs which respond to similar cellular signalling pathways which are dependent on chromatin structure.

Primary DNA sequence which contains stretches of tandem guanine nucleotides can fold into four-stranded structures called G4 DNA, which are implicated in gene expression, replication and telomere maintenance [21]. The presence of G4 sequences along with abnormal hypomethylation was shown to be enriched in breakpoints mapped in cancer genomes, leading to the hypothesis that loss of methylation in regions with G4 sequences is part of the mutagenic processes in cancer [25]. Computational analyses using such as the Quadparser programme have suggested these structures are prevalent in the human genome with data demonstrating their function *in vitro*[23,26]. SVAs contain sequences with G4 potential, specifically in their CCCTCT hexamer and central VNTR (Figure 2), therefore could show similar properties to already characterised functions of G4 DNA mentioned previously. Of particular interest would be the hypothesised mutagenic properties of G4 sequences in demethylated regions in cancer as it has been demonstrated that SVAs experience a loss of methylation in cancer [19]. The amount of G4 potential and the domain of the SVA it was predominantly located in varied across the different subtypes. The older subtypes (A, B and C) had the lowest potential; which was mostly located within the 5’ CCCTCT repeat, whereas the younger human specific (E, F and F1) demonstrated the greatest potential for G4 with an increase in the amount located in the central VNTR. Subtype D showed itself to be an intermediate of the two groups.

The polymorphic nature of SVAs extends to their presence or absence in the genome, this has been analysed for a group of human specific SVAs, it was estimated that 37.5% of SVA Es and 27.6% of SVA Fs were polymorphic in their occurrence in the genome [5]. The frequency of this presence or absence of specific SVAs located in *HLA* genes has shown to be variable between groups with different ethnic origins [53]. This demonstrates the variability of SVA insertions between individuals; our study extends the analysis of their polymorphic nature to include the variation found in the CCCTCT hexamer repeat and provides further evidence of the already characterised variation in the second domain of the central VNTR (Table 2). Our data demonstrates the *PARK7* SVA has at least four alleles which show variation in the two regions above, which interestingly are also the major regions for potential G4 DNA.

The final parameter we explored was the potential for the SVA to act as a transcriptional regulator in a classical reporter gene model (Figure 4). Although this assay did not allow us to address epigenetic parameters it did allow us to address whether the primary sequence of the SVA could interact with transcription factors to modulate transcriptional properties and further allowed us to delineate potential distinct regulatory domains in the SVA. The definition of the latter was particularly important given the accepted composite nature of domains in SVAs; tandem repeat structures are a class of regulatory DNA which we and others have demonstrated can direct tissue specific and stimulus inducible expression *in vitro* and *in vivo* both in mammals and herpes simplex virus [31,35,54]. We focused our analysis on the human specific SVA in the promoter of the *PARK7* gene. As shown in Figure 4B and C the central TR/VNTR differentially supported reporter gene expression in the two cell lines analysed. It demonstrated repressive qualities in the neuroblastoma cell line SK-N-AS but not in the breast cancer cell line MCF-7 when in the forward orientation. These cell lines were selected they are well characterised and accepted to represent neuronal function (SK-N-AS) and breast cancer (MCF-7) because *PARK7*, also termed *DJ-1*, is associated with both breast cancer and early onset Parkinson’s disease [55,56], further they provide preliminary functional data on the ability of the *PARK7* SVA to affect expression in different environments. We have previously shown that VNTRs can function in a tissue specific manner so the distinct functions in the cell line models were not unexpected.

The complete SVA showed no activity in the SK-N-AS cell line but enhanced reporter gene expression in MCF-7 cells. Interestingly the deletion of the SINE element from the SVA fragment resulted in significantly higher levels of reporter gene expression than the SVA alone in both cell lines. This leads us to postulate that there are probably a minimum of three distinct functional elements in the SVA that adjust its ability to modulate expression, the central TR/VNTR, SINE and the CCCTCT and Alu-like sequences. The data on the central TR/VNTR indicated they support distinct transcriptional properties dependent on cell type. This is consistent with the action of VNTRs we have previously observed in the human serotonin and dopamine transporter genes [28,31,34]. We would expect that different complements of transcription factors present in both these cell lines are responsible for the activity of the reporter gene directed by the TR/VNTR.

## Conclusions

We propose that SVAs have inserted preferentially into genic regions placing them in areas of the genome where they have the potential to affect transcription or post transcriptional regulation through several mechanisms such as methylation state, provision of multiple transcription factor binding sites or formation of DNA secondary structures. We studied the *PARK7* SVA in detail, demonstrated its ability to differentially affect transcription within a reporter gene construct in two different cell lines and identified at least four alleles for this particular SVA with multiple regulatory domains. We and others have previously demonstrated the functional consequences, transcriptional properties or utilisation as a biomarker in the human genome for both mental health and cancer of VNTRs. Therefore mechanistically the polymorphic variation we observed can potentially affect several parameters. We also demonstrated *in silico* that the CCCTCT and central VNTR domains have the potential to form distinct secondary structures (G4), which impart function. There was an increase in the amount of G4 potential, in particularly in the central VNTR, as the SVAs progressed to the younger human specific subtypes as changes occurred in their structure and sequence.

## Methods

### Analysis of distribution and structure of SVAs

A list of SVAs from the repeat masker track of UCSC genome browser (http://genome.ucsc.edu/index.html) with Hg19 was generated and then manually annotated to include any components of the SVA that had not been included. This list along with the UCSC table browser and Galaxy software (https://main.g2.bx.psu.edu/) was used to analyse the distribution of SVAs across the genome. The size and gene content of each chromosome was taken from NCBI human genome overview for 37.3. Quadparser software (http://www.quadruplex.org/) was used to predict the potential of the SVA sequence to form G4 DNA.

### Cell culture

Complete media for SK-N-AS cell line: Dulbecco’s Modified Eagles medium with 4500 mg glucose/L (Sigma) supplemented with 1% (v/v) non essential amino acid solution (Sigma), 100 units per ml of penicillin and 0.1 mg/ml of streptomycin and 10% (v/v) foetal bovine serum (Sigma). Complete media for MCF-7 cell line: Dulbecco’s Modified Eagles medium with 4500 mg glucose/L (Sigma) supplemented with 100 units per ml of penicillin and 0.1 mg/ml of streptomycin and 10% (v/v) foetal bovine serum (Sigma). Cells were grown in 5% CO_2_ and at 37°C.

### Cloning of *PARK7* SVA fragments into pGL3P

The three fragments of the *PARK7* SVA; SVA, SVA wo SINE and the TR/VNTR of the SVA, were amplified using PCR with KOD Hot Start Polymerase (Novagen) under standard conditions with the following primers sets respectively: 5’GGCTTTTTGATAACCCCTGA 3’ and 5’TTTCGGATCACAGGCATGAGC 3’, 5’ GGCTTTTTGATAACCCCTGA 3’ and 5’ CCGCCTTTCTATTCCACAAA

3’, 5’CTCAGTGCTCAATGGTGCC 3’ and 5’ CCGCCTTTCTATTCCACAAA 3’. JAr genomic DNA was used as template to amplify the whole SVA and the SVA without the SINE region. The whole SVA amplicon was used in nested PCR to amplify the TR/VNTR of the SVA. These three fragments were sub cloned into an intermediate vector (Zero Blunt PCR vector from Invitrogen) and sequence confirmed by DNA Sequencing and Services, University of Dundee, the fragments corresponded to allele *1*. During this cloning process a truncated SVA was generated during one of the transformation steps and this 5’ fragment was used to produce a fourth reporter gene construct. These intermediate plasmids were firstly digested with restriction enzymes Acc65I and XhoI (Promega) and inserts cloned into the multiple cloning site of pGL3P reporter gene vector upstream of the SV40 minimal promoter (Promega) so that all inserts were in the forward orientation (CCCTCT hexamer at 5’ end and poly A-tail at 3’ end) and secondly digested with the restriction enzymes BamHI and XbaI, and cloned in to the multiple cloning site of pGL3P which had been digested with Nhe1 and BglII. This resulted in the generation of reporter gene vectors containing the *PARK7* SVA fragments in reverse orientation (poly A-tail at 5’ end and the CCCTCT hexamer at the 3’ end).

### Transfection of reporter gene constructs and luciferase assay

The cells were plated out in 24 well plates at the following concentrations 24 hrs prior to transfection: SK-N-AS 120,000 cells per well and MCF-7 100,000 cells per well. Reporter gene constructs (1 μg) and internal control TK renilla construct (10 ng) used for normalisation of data, were co transfected using TurboFect (Thermo Scientific) following manufacturers’ instructions. Cells were lysed 48 hrs post-transfection and the Dual Lucificerase Reporter Assay (Promega) was performed, luminescence was measured with a Glomax 96 Microplate Luminometer (Promega). Statistical analysis to test the significance of the fold change of the reporter gene constructs over the minimal promoter alone and comparison of forward and reverse orientation fold activity were carried out using a one tailed *t*-test. Significance was scored as follows * P < 0.05, **P < 0.01, ***P < 0.001 and #P < 0.05, ##P < 0.01, ###P < 0.001 N = 4.

### Genotyping *PARK7* SVA

The *PARK7* SVA was amplified using the following primer set: forward 5’GGCTTTTTGATAACCCCTGA 3’ and reverse 5’GCAAGGCTTAGCTTGGACAG 3’ and KOD Hot Start DNA Polymerase (Novagen) under standard conditions with the addition of betaine (Sigma) at 0.5 M final concentration. 1 ng of genomic DNA from the CEU HapMap cohort was used as template. The PCR products were run on 1% agarose gels stained with GelRed Nucleic Acid Stain (Biotium) and visualised using a UV transilluminator (BioDoc-it Imaging System). Alleles that were difficult to call were repeated and any that remained ambiguous were excluded.

## Abbreviations

SVA: SINE-VNTR-Alu; LTRs: Long terminal repeats; LINEs: Long interspersed elements; SINEs: Short interspersed elements; VNTR: Variable number tandem repeat; TR: Tandem repeat; G4: G-quadruplex.

## Competing interests

The authors declare no competing interests.

## Authors’ contributions

All authors (ALS, VJB, GB and JPQ) contributed to the design, analysis and interpretation of the study. ALS performed the bioinformatic analysis, reporter gene assays and genotyping. ALS and GB completed statistical analysis. All authors contributed to the production of the manuscript. All authors read and approved the final manuscript.

## Supplementary Material

Additional file 1 **Gene and SVA density of human chromosomes (.pdf).** Data values for graph in Figure 1A showing the SVA and gene densities for each individual chromosome. Click here for file

Additional file 2 **Correlation coefficient of gene and SVA subtype density across human chromosomes (.pdf).** A table showing the correlation coefficients between each SVA subtype density and gene density of human chromosomes. Click here for file

Additional file 3 **Distribution of SVA subtypes within 1 kb, 10 kb, 20 kb and 100 kb upstream of a transcriptional start site (.pdf).** A graph comparing the distribution of each SVA subtype in defined regions upstream of transcriptional start sites to their distribution across the whole genome. Click here for file
