# Supplementary material for: Characterisation of the potential function of SVA retrotransposons to modulate gene expression patterns
Source: BMC Evol Biol. 2013 May 21;13:101. doi: 10.1186/1471-2148-13-101 (PMC3667099; doi:10.1186/1471-2148-13-101)
Supplement: Additional file 1 — Gene and SVA density of human chromosomes (.pdf). Data values for graph in Figure 1A showing the SVA and gene densities for each individual chromosome. [file 1471-2148-13-101-S1.pdf]

Additional file 1

Gene and SVA density of human chromosomes

Chromosome	Gene Density	SVA Density
1	14.09	1.07
2	9.74	0.84
3	6.54	0.89
4	7.55	0.70
5	9.03	0.82
6	12.02	0.85
7	11.83	0.85
8	8.98	0.67
9	10.86	0.79
10	10.26	0.89
11	16.06	0.99
12	12.81	0.94
13	6.25	0.56
14	14.27	0.80
15	12.18	0.71
16	14.68	0.89
17	21.84	1.45
18	7.13	0.65
19	34.94	2.01
20	14.14	1.17
21	9.35	0.42
22	16.67	0.84
X	10.77	0.88
Y	7.23	0.20

Values for graph in figure 1A showing the gene density and SVA density for each chromosome as number per million bases.
