# Supplementary material for: Characterisation of the potential function of SVA retrotransposons to modulate gene expression patterns
Source: BMC Evol Biol. 2013 May 21;13:101. doi: 10.1186/1471-2148-13-101 (PMC3667099; doi:10.1186/1471-2148-13-101)
Supplement: Additional file 2 — Correlation coefficient of gene and SVA subtype density across human chromosomes (.pdf). A table showing the correlation coefficients between each SVA subtype density and gene density of human chromosomes. [file 1471-2148-13-101-S2.pdf]

Additional file 2

Correlation coefficient of gene and SVA subtype density across human chromosomes

SVA Subtype	Correlation Coefficient
A	-0.55
B	0.32
C	0.59
D	0.72
E	0.36
F	0.70
F1	0.31

Correlation coefficients for the relationship of SVA density with gene density for each SVA subtype calculated using the bootstrap confidence interval (95%) to remove outliers. All subtypes showed a positive relationship between the two variables except A.
