# Supplementary material for: Characterisation of the potential function of SVA retrotransposons to modulate gene expression patterns
Source: BMC Evol Biol. 2013 May 21;13:101. doi: 10.1186/1471-2148-13-101 (PMC3667099; doi:10.1186/1471-2148-13-101)
Supplement: Additional file 3 — Distribution of SVA subtypes within 1 kb, 10 kb, 20 kb and 100 kb upstream of a transcriptional start site (.pdf). A graph comparing the distribution of each SVA subtype in defined regions upstream of transcriptional start sites to their distribution across the whole genome. [file 1471-2148-13-101-S3.pdf]

## Additional file 3

**Distribution of SVA subtypes within 1kb, 10kb, 20kb and 100kb upstream of a transcriptional start site**

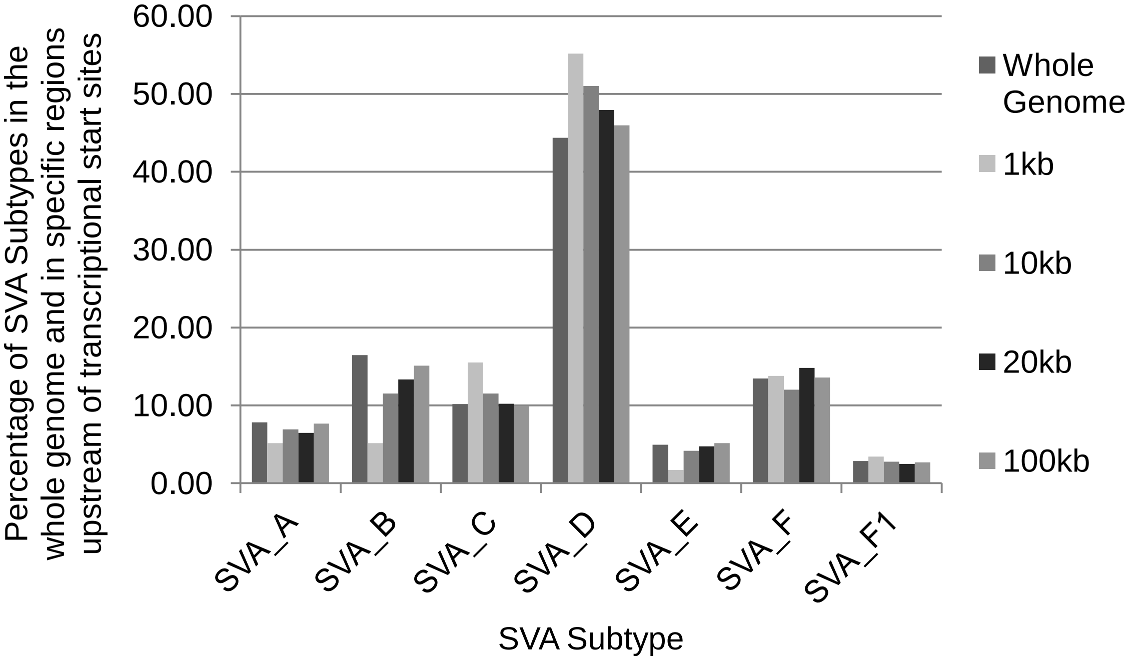


The distribution of SVA subtypes within regions upstream of a transcriptional start site. There is a significant difference in the distribution of the subtypes within the first kilobase compared to the whole genome. (1kb $X^2$=16.30, df=6, $p<0.05$), (10kb $X^2$=2.97, df=6, p=0.81), (20kb $X^2$=1.30, df=6, p=0.97), (100kb $X^2$=0.20, df=6, p=0.99).
